# What have we learned from global change manipulative experiments in China? A meta-analysis

**DOI:** 10.1038/srep12344

**Published:** 2015-07-24

**Authors:** Zheng Fu, Shuli Niu, Jeffrey S. Dukes

**Affiliations:** 1 Key Laboratory of Ecosystem Network Observation and Modeling, Institute of Geographic Sciences and Natural Resources Research, CAS, Beijing 100101, China; 2 University of Chinese Academy of Sciences, No. 19A Yuquan Road, Beijing, 100049, China; 3 Department of Forestry and Natural Resources, and Department of Biological Sciences, Purdue University, West Lafayette, IN, USA

## Abstract

Although China has the largest population in the world, a faster rate of warming than the global average, and an active global change research program, results from many of the global change experiments in Chinese terrestrial ecosystems have not been included in global syntheses. Here, we specifically analyze the observed responses of carbon (C) and nitrogen (N) cycling in global change manipulative experiments in China, and compare these responses to those from other regions of the world. Most global change factors, vegetation types, and treatment methods that have been studied or used elsewhere in the world have also been studied and applied in China. The responses of terrestrial ecosystem C and N cycles to N addition and climate warming in China are similar in both direction and intensity to those reported in global syntheses. In Chinese ecosystems as elsewhere, N addition significantly increased aboveground (AGB) and belowground biomass (BGB), litter mass, dissolved organic C, net ecosystem productivity (NEP), and gross ecosystem productivity (GEP). Warming stimulated AGB, BGB and the root-shoot ratio. Increasing precipitation accelerated GEP, NEP, microbial respiration, soil respiration, and ecosystem respiration. Our findings complement and support previous global syntheses and provide insight into regional responses to global change.

Over the last century, anthropogenic activities have increased the atmospheric CO_2_ concentration and N deposition rates, leading to climatic changes like warming and precipitation changes^1^. These unprecedented global changes have greatly affected ecosystem functions and services^2^,^3^. Ecosystem C and N cycles, two of the most important biogeochemical cycles that have been largely influenced by global change, also feedback and regulate climate change magnitude^4^,^5^. To better understand the interactions between terrestrial ecosystems and global change, scientists have conducted hundreds of global change experiments around the world^6^,^7^,^8^. While individual experiments advance our understanding of the responses of specific ecosystems to global changes, the general patterns of responses at regional and global scales are very useful for benchmarking regional and global models and informing policy related to sustaining ecosystem services or enhancing the ability of local communities to adapt to global change^9^,^10^.

China has the world’s largest population, whose activities have accelerated the changes in climate and ecosystem processes^2^. According to the Third National Assessment Report on Climate Change^11^, annual average air temperature in China has increased by 0.9–1.5 °C during the past 100 years and will increase by 1.3–5 °C by the end of this century, which is larger than the average global temperature rise. Annual precipitation has also changed considerably with increasing precipitation in semiarid areas in the past 30 years. The coastal sea level has increased by 2.9 mm/year from 1980–2012 in China^11^. Moreover, Chinese ecosystems have high spatial heterogeneity and diverse biogeography. China’s wide ranges of latitude and longitude, and topography, which includes the Qinghai-Tibetan Plateau (Earth’s ‘third pole’, average elevation of 4000 m a.s.l.), create complex and diverse ecosystem types and vegetation communities^12^,^13^. With its wide variation in climatic conditions and ecological communities and large scale of ongoing and expected human disturbance, China provides a good platform for studying ecosystem responses to global changes at the regional scale. Although many manipulative experiments have been conducted in China, few have been included in global syntheses^14^,^15^,^16^,^17^,^18^,^19^,^20^ (Supplementary Figs S1, S2), largely because most experimental studies were published in non-English language journals.

In this study, we review the types of global change manipulative experiments that have been conducted in China and their distribution across ecosystem types, and analyze the responses of ecosystem C and N cycles to major global change drivers. Specifically, we: 1) provide an overview and database of global change manipulation experiments in China; 2) use meta-analysis to summarize global change responses of properties and processes relevant to the C and N cycles in China’s ecosystems, and 3) compare the responses of China’s ecosystems to warming and N addition with those from previous global meta-analyses of responses of the C and N cycles to warming and N addition.

## Results

### Global change manipulation experiments in China

**Figure 1:**
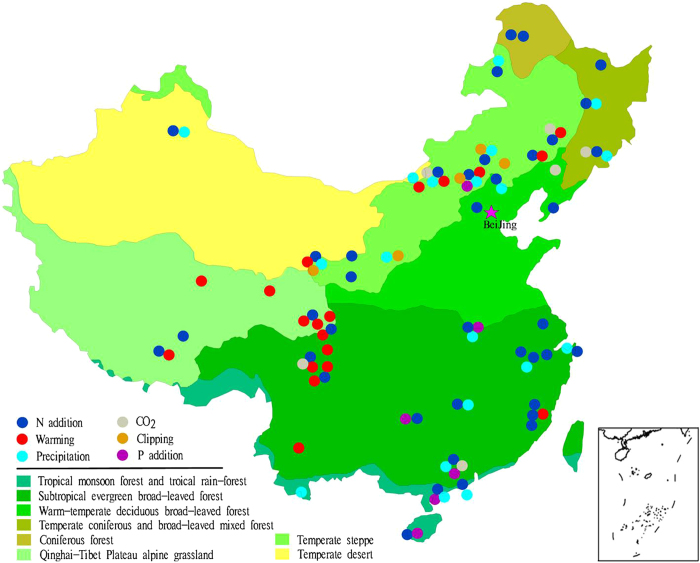
The number of observations in different experiments of N addition, warming and altered precipitation in China. The map was generated from the Compilation Group of Vegetation Atlas of China (1:1000000)^50^. The software MapGIS 6.7 (Zondy Cyber Group Co., Ltd. Wuhan, China) was used to create the map[s].

We identified 94 global change manipulation experiments that were conducted in China (Fig. 1, Supplementary Table S1). The treatments most frequently manipulated were nitrogen addition, warming, altered precipitation, elevated atmospheric CO_2_, clipping the grassland to simulate grazing or hay production, and phosphorus addition. We identified 39 experiments with N addition, 19 with warming, 19 with altered precipitation, and 17 with other factors (e.g. CO_2_, phosphorus addition; Fig. 1). We did not conduct further research on the results from these latter 17 experiments, as there were too few results from each type of manipulation to conduct reliable meta-analyses.

**Figure 2:**
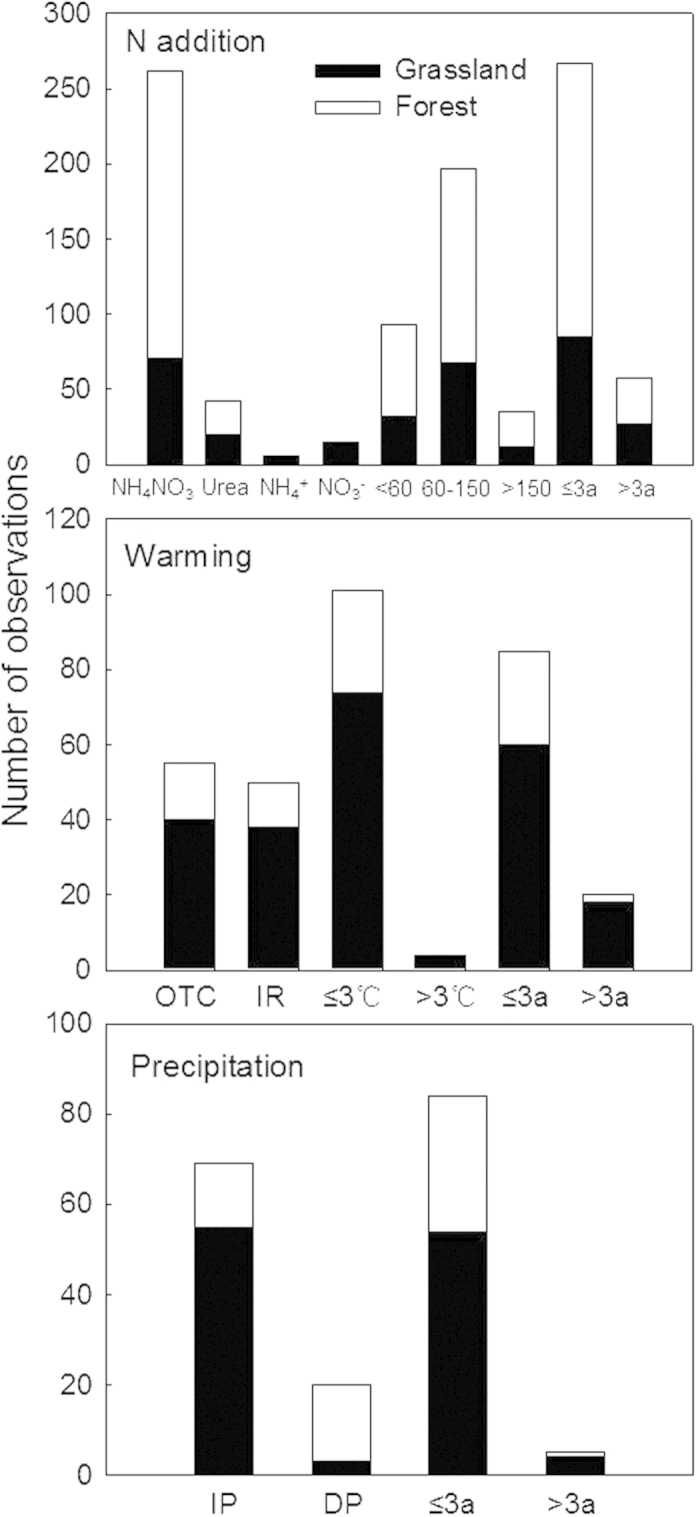
The number of observations in different experiments of N addition, warming and altered precipitation in China. (**a**) warming (**b**) and altered precipitation (**c**) experiments in China. <60 means that the nitrogen addition rate was <60 kg N ha^−1^ yr^−1^. 3a means the experiment lasted for 3 years. OTC: open top chamber, IR: infrared radiation, IP: increased precipitation, DP: decreased precipitation.

The distribution of experiments across ecosystem types differed among N addition, warming and altered precipitation experiments. For N addition experiments, 25 were conducted in forests and 14 in grasslands. The vegetation types studied were mainly subtropical evergreen broad-leaved forest, temperate steppe, warm temperate deciduous broad-leaved forest and temperate mixed broadleaf-conifer forest (Fig. 1). Nitrogen was mostly applied as NH_4_NO_3_ (262 observations, 71 in grassland and 191 in forest). Urea, NH_4_^+^, and NO_3_^−^ were applied less frequently. Nitrogen was most frequently added at the relatively high rates of 60–150 kg ha^−1^yr^−1^, and most studies lasted no more than 3 years (Fig. 2a).

Warming experiments were conducted mainly in temperate steppe, subtropical evergreen broad-leaved forest, and alpine grassland in the Tibetan Plateau (Fig. 1). Only 6 of these experiments took place in forests, presumably because of the technical challenges and high cost involved with warming forest. Thirteen of the experiments were conducted in grasslands. Open-top chambers and infrared heaters were the most common warming technologies, accounting for 55 and 50 observations, respectively. Moreover, most of the experiments were warmed 0–3 °C and most published studies were conducted for no more than 3 years (Fig. 2b).

Effects of precipitation change were most commonly studied in temperate steppe and subtropical evergreen broad-leaved forest (Fig. 1). Eleven of the precipitation manipulation experiments were conducted in forests and 8 in grasslands. Experiments that increased precipitation were conducted mostly in grassland while those decreasing precipitation were conducted mostly in forest (Fig. 2c). Furthermore, the precipitation treatments differed among experiments, with some studies changing precipitation percentage, while others changing precipitation amount.

### Comparison to global syntheses

#### N addition experiments

**Figure 3:**
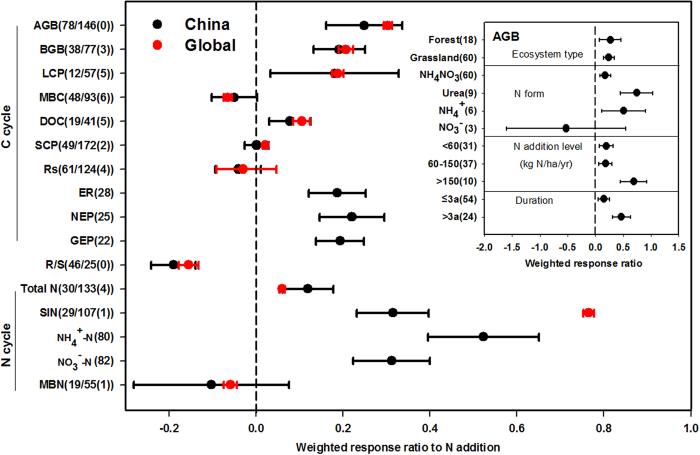
Comparisons of the responses of ecosystem C and N variables to N addition in China and at the global scale. The insert is the response ratio of AGB in different ecosystems in China and with different nitrogen forms, rates and experimental durations. Bars represent 95% confidence intervals. The vertical lines are drawn at In RR = 0. The sample size for each variable is shown in parentheses (Sample size for Chinese study/sample size for global study (Chinese results included in global study)). See abbreviations in Table 1.

Most of the response variables studied in global change experiments were ecosystem C and N cycles. So, we compared the weighted response ratios observed in China (RR_China_) with those from previous global analyses (RR_global_) for 16 variables related to ecosystem C and N cycles, examining changes in both direction and intensity. Few of the experiments in China had been included in the global syntheses (Fig. 3). Overall, N addition increased both above- and below- ground biomass (AGB, BGB) in China and the globe, with RR_China_ of 0.25 and 0.19 and RR_global_ of 0.30 and 0.21, respectively, and an associated decline in root-shoot ratios (RR_China_ of −0.19, RR_global_ of −0.16) (Fig. 3). Nitrogen addition also significantly stimulated litter C and dissolved organic C (DOC). Microbial biomass C (MBC) and the soil C pool (SCP) exhibited no significant changes (Fig. 3). In China, N addition increased ecosystem respiration (ER), net ecosystem production (NEP) and gross ecosystem production (GEP), with RR_China_ of 0.19, 0.22 and 0.19, respectively (Fig. 3), but we did not find any meta-analysis results at the global scale. Nitrogen addition did not significantly change soil respiration (Rs) in either China or the globe, but did significantly increase soil N. In China, soil total N, inorganic N (SIN), NO_3_^−^-N, and NH_4_^+^-N concentration all increased, with RR_China_ values of 0.12, 0.31, 0.52 and 0.31, respectively (Fig. 3). At the global scale, soil total N and SIN increased by RR_global_ of 0.06 and 0.77, respectively. Microbial biomass N (MBN) did not change with external N input. Out of the 11 variables that had response ratios for both China and the globe, SIN was the only variable for which RR_china_ significantly differed from RR_global_.

For the variables (e.g., AGB) that had a large enough sample size, we compared RR_China_ between ecosystem types, N addition forms and rates, and experimental durations (Fig. 3). Nitrogen addition significantly increased AGB in both forest and grassland. Urea addition increased AGB more than the addition of NH_4_NO_3_ and NH_4_^+^-N. The effects of NO_3_^−^-N addition varied widely, in part because only three studies included this treatment. The highest N addition rates (>150 kg N ha^−1^yr^−1^) increased AGB more than lower N addition rates (<60 or 60–150 kg N ha^−1^yr^−1^). RR_China_ was larger in long-term than short-term experiments (Fig. 3).

#### Warming experiments

**Figure 4:**
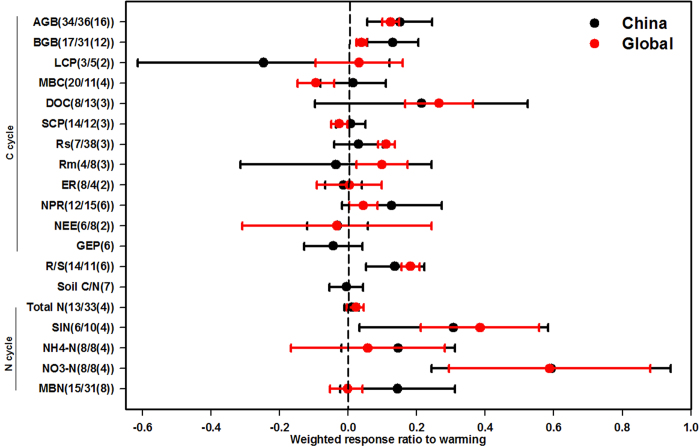
Comparisons of the responses of ecosystem C and N variables to warming in China and at the global scale. The sample size for each variable is shown in parentheses (Sample size for Chinese study/sample size for global study (Chinese results included in global study)). See abbreviations in Table 1.

In aggregate, warming responses observed in Chinese experiments resembled those of the global population of experiments, although few of the results in China were included in the global syntheses (Fig. 4). Specifically, AGB and BGB were significantly enhanced by warming, with RR_China_ of 0.15 and 0.12, respectively (Fig. 4). Warming increased the root-shoot ratio for both RR_China_ (0.14) and RR_global_ (0.18) (Fig. 4). However, experimental warming did not affect the litter C pool either in China or at the global scale. The RR_China_ of MBC, DOC, SCP, Rs, microbial respiration (Rm) and net photosynthetic rate were not significantly different from RR_global_, but the changes in MBC, DOC, SCP, Rs, Rm and net photosynthetic rate were significant at the global scale while not in China. ER and NEE did not significantly change with warming either in China or at the global scale. Of the N cycle variables, warming significantly stimulated SIN and soil NO_3_^−^-N concentration for RR_China_ (0.31 and 0.59) and RR_global_ (0.39 and 0.59), respectively (Fig. 4), but soil total N concentration, soil NH_4_^+^-N and MBN concentration showed no significant responses to experimental warming either in China or at the global scale. We did not find meta-analysis results of GEP and soil C:N at the global scale, and these variables exhibited no significant response to warming in China. Due to the limited sample size for warming experiments, we did not separate the results into different ecosystem types, treatment methods or experimental durations.

#### Precipitation change experiments

**Figure 5:**
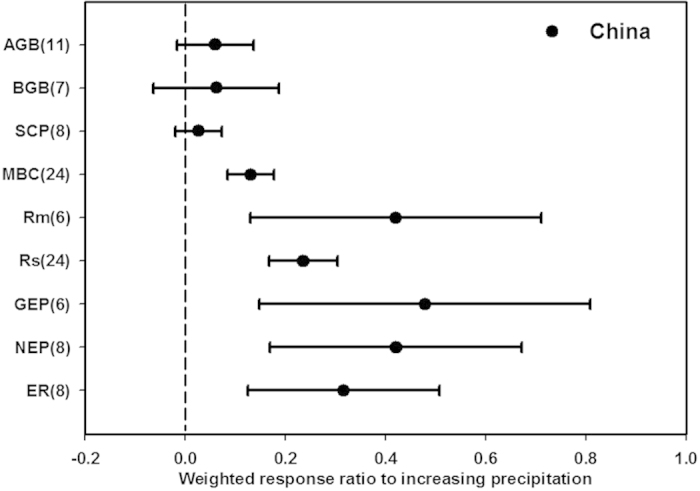
The responses of ecosystem C variables to increasing precipitation in China. See abbreviations in Table 1.

Conducting meta-analyses for altered precipitation is much more complicated than doing such analyses for N addition and warming experiments, because of the large variations in treatment types and many mediating factors such as soil texture and rooting depth that may influence organisms’ responses to precipitation. We did not find any global studies with response ratios for precipitation. Wu *et al.* (2011) conducted a meta-analysis on the interactions of warming and precipitation change, but indicated the responses by sensitivity rather than response ratio^20^. Our analysis showed that increased precipitation treatments enhanced Rm, Rs, GEP, NEP and ER, for mean RR_China_ of 0.42, 0.24, 0.48, 0.42, and 0.32, respectively (Fig. 5). Increasing precipitation also significantly stimulated MBC for RR_China_ of 0.13. However, AGB, BGB and SCP showed no significant changes (Fig. 5). Although we could not compare these values to RR_global_, the response directions of terrestrial ecosystems to precipitation change in China were approximately consistent with the global-scale results^20^.

## Discussion

### Global change experiments in China

In China, modernization in recent decades has made available capital to invest in science, but also brought a rapid deterioration of environmental conditions. The former makes research possible and the latter makes ecological research necessary. As a consequence, ecology has expanded rapidly in China, and a large number of global change experiments have been conducted over the last decade.

At least 94 global change manipulation experiments have been conducted in China. However, results from most of these studies have only been published in Chinese-language journals, and only 3% of the results in N addition experiments and 5% of the results in warming experiments in China were included in global syntheses (see Supplementary Figs S1, S2). The global change manipulations conducted in China mainly include N addition, warming, and precipitation change. Some types of experiments, like warming forest ecosystems, decreasing precipitation, increasing CO_2_, or adding phosphorus have rarely been conducted in China (Fig. 1). To more fully understand responses of Chinese ecosystems to global change, these types of experiments should be encouraged, and ideally conducted in ecosystems with the greatest potential to feed back to global changes^7^,^21^.

The vegetation types studied mostly were subtropical evergreen broad-leaved forest and temperate steppe. In China, relatively few global change manipulations have been conducted in the unique tropical monsoon, rain-forest regions, or the Tibetan Plateau. We recommend that future experiments focus on these systems, which have large carbon uptake capacity or soil carbon storage^22^,^23^,^24^, and are sensitive to global change^25^,^26^,^27^,^28^. The lack of experiments in these critical areas leaves many hypotheses and model predictions untested.

Many of the methods that have been used to apply global change treatments elsewhere in the world have also been employed in China. Our analysis suggests that different N addition forms and amounts have different effect magnitudes (Fig. 3), which makes it difficult to compare across all types of N addition experiments. Moreover, it is a challenge to synthesize across precipitation manipulations, because the magnitudes of treatments (e.g. −20% precipitation, plus 120 ml, double or half precipitation) often do not clearly indicate the actual amount of precipitation manipulated^20^, and because changes in the amount of incoming precipitation are less important to the ecosystem than changes in the amount of water that plants can access^29^. To make experiments more comparable, some scientists have called for coordinated manipulative experiments that use standardized research designs and approaches to address similar scientific questions across broader geographic areas^6^,^29^,^30^. Since the research results from China often largely reflect the global response patterns, establishing a series of long-term experiments with a systematic, universally standardized methodology accounting for global change factors in China would help to better understand terrestrial ecosystem responses to global change.

Most global change experiments in China were conducted for no more than 3 years, especially the precipitation change experiments. However, ecosystem responses to global change are strongly regulated by long-term processes^7^,^29^. Understanding of long-term processes is essential to test and constrain models in order to more realistically predict ecosystem responses across longer time scales. We recommend that funding agencies and governments give special consideration to support for long-term experiments that address global change issues in China.

### Responses in China resemble global responses

The results from this meta-analysis indicate that the responses of ecosystem C and N cycles to N addition and warming in China are approximately consistent with those at the global scale in both response direction and intensity (Figs 3 and 4). For example, for C cycle process, our meta-analysis results show that N addition significantly increased AGB, BGB, the litter C pool, DOC, NEP and GEP, and decreased the root-shoot ratio. Similar results have been reported in global syntheses of N addition^17^. Warming stimulated AGB, BGB and the root-shoot ratio in both Chinese ecosystems and global syntheses^18^,^19^, but we note the global syntheses of these variables already had good representation from Chinese experiments. In Chinese ecosystems, increased precipitation accelerated GEP, NEP and other C fluxes (microbial respiration, soil respiration and ecosystem respiration), suggesting that C cycling is highly sensitive to increased precipitation. These results also parallel those of a global synthesis^20^. The responses of terrestrial ecosystems to N addition, warming, and increasing precipitation as revealed by this synthesis could be potentially useful for parameterizing and benchmarking ecosystem models for predicting Chinese ecosystem responses and feedbacks to global change.

Why did results from experiments in China resemble those from global syntheses? First, the latitudinal distribution of global change experiments in China was similar to that of experiments in global analyses (Supplementary Figs S1, S2). Because similar latitudes are often climatically similar, they often have similar ecosystem types and vegetation communities^2^,^31^. Second, the experimental methods used in China were similar to those used elsewhere. For example, the forms of N addition applied in China were consistent with those found in global meta-analyses^15^,^17^. Likewise, open top chambers and infrared heaters were the most common technologies used in warming experiments, both in China and globally. The magnitude of warming in most experiments was 0–3 °C^18^, and experimental durations in China were similar to those elsewhere. Although more experiments were encompassed in global-scale analyses, the proportion of experiments of short duration was approximately consistent in the two scales of analyses. Third, the similar responses of ecosystem C and N cycles to global change drivers in China and elsewhere may also result from China’s high geographic heterogeneity and complex topography, which give rise to a wide variety of climatic zones and vegetation types. The similarity of results between China and globe suggest that we may develop a globally consistent strategy in evaluating terrestrial responses and adapting to or mitigating climate change.

Only a few variables responded differently in the Chinese and global syntheses. The response ratio of soil inorganic N to N addition was significantly lower in the Chinese ecosystems than in the global synthesis (Fig. 3), possibly because the average amount of N applied in Chinese experiments that studied soil inorganic N was substantially less (7.3 gN m^−2^ yr^−1^) than the average from the global synthesis (13.3 gN m^−2^ yr^−1^)^16^. The changes in MBC, DOC, SCP, Rs, Rm and net photosynthetic rate were significant at the global scale, but not in China (Fig. 4). Some of these differences were probably a consequence of the smaller sample size and thus larger variation in the dataset from China. However, differences among methodologies, biomes, and environmental conditions likely also contributed to these differences^32^,^33^,^34^.

### Implications and the way forward

Our synthesis suggests that global changes will increasingly affect ecosystem C and N cycles in China. If N deposition and warming continue as projected, our results indicate that AGB and BGB will increase. Ecosystem C sequestration may increase under N deposition, but not change under climate warming (Figs 3 and 4). However, the large variation in the response ratios indicates that impacts vary greatly across vegetation types and/or temporal scales. A more adequate comparison of responses across China’s heterogeneous landscapes and vegetation communities would require a more comprehensive suite of experimental studies. To better understand the mechanisms underlying the responses of ecosystem C and N cycles to climate change, more related processes such as plant and microbial community structure and other biogeochemical cycles need to be more extensively studied. Moreover, to improve our predictive understanding of climate and ecosystem functioning, experimental results from these studies need to be used as benchmarks to constrain regional or global models^9^.

Despite its focus on China, this study has implications for global change studies in other regions of the world. Our results indicate that integration of studies published in non-English journals into global meta-analysis could potentially reduce variation in global syntheses by increasing sample sizes or degrees of freedom^35^. Previous meta-analyses that quantified responses of terrestrial ecosystem C and N cycles to N addition^15^,^16^,^17^, climate warming^14^,^18^,^19^, and precipitation changes^20^ primarily compiled data published in international, English-language journals, omitting studies published in other languages and underemphasizing results from non-English speaking countries. Policy makers in those countries, however, may rely on results published in the local journals to make policy decisions. The findings in this synthesis complement global syntheses, increase awareness of the wide range of Chinese global change experiments that have been conducted in recent years, and provide insight into regional responses to global change. It is a challenge to assemble all of the results from global change experiments throughout the world. By setting an example in this study, we suggest that, particularly for non-English-speaking countries, country-level databases can help to disseminate data more widely, allowing more complete use of available data to increase our understanding of the natural world.

## Methods

### Data compilation

In this study, peer-reviewed papers published before 2014 were searched from ISI Web of Science, Google Scholar (Google Inc., Mountain View, CA, USA), and the China Knowledge Resource Integrated Database (*available online*: http://www.cnki.net/). The searches looked for studies with keywords, titles or abstracts related to: global change experiment, China, N addition/deposition/input/application/fertilization/enrichment, warming/increasing temperature, altering/changing precipitation, increasing/decreasing precipitation, irrigation, watering, elevated/increased CO_2_, phosphorus addition/deposition/input/application/fertilization/enrichment, and clipping. The literatures list showed that a large number of global change manipulative experiments have been conducted in China (Fig. 1, Table S1). We selected finally 118 papers into our meta-analysis (see Supplementary Text and Supplementary Database), and papers had to meet all of the following criteria: (i) The experiment was conducted in China, and included at least one of our selected variables. (ii) Experimental treatments were applied to the plots in the field in natural ecosystems with no management, mainly forest and grassland ecosystems. (iii) The methods used in the experiment and the experimental duration were clearly indicated. (iv) The means, standard deviations or standard errors, and sample sizes of the variables were directly reported or could be calculated from the papers. It should be noted that measurements for different treatment levels (e.g. N application rates, warming magnitudes and increasing precipitation amount) were considered as independent observations if more than one level was applied in the same experiment^15^,^18^. For multifactor studies, we used the data for each pair of combined treatment vs. ambient treatments^20^,^36^. For example, for the experiments that included the four treatments of control, warming, nitrogen addition, and warming plus nitrogen addition, we calculated two warming response data points by comparing warming vs. control, and warming plus nitrogen addition vs. nitrogen addition. Including multiple results from a single study violates the assumption of independence in meta-analysis^37^,^38^. Therefore some researchers have advocated the inclusion of only one result from each study^15^,^18^,^37^,^39^,^40^ when considering the lack of independence to be a serious problem for meta-analysis. However, the loss of information caused by the omission of multiple results in each study may become a more serious problem than that caused by violating the assumption of independence^41^. Thus, many researchers have included more than one result from a single study in their meta-analyses^36^,^42^,^43^,^44^,^45^. Different experiments (different started time and/or different treatment methods) for same manipulation factors, conducting in the adjacent areas, were included in our analysis. To avoid losing information of these experiments, we used averaged sampling for every year. To compare the meta-analysis results in China with those from the global scale, we extracted the global response ratios directly from individual meta-analysis papers. We chose the global meta-analysis papers that were published most recently and covered the most experimental studies, climate areas, and vegetation types^14^,^16^,^17^,^18^,^20^.

The compiled database included 19 variables associated with ecosystem C and N cycles (Table 1), including aboveground biomass, belowground biomass, the litter C pool, and other variables, and their responses to global change factors. We only compiled databases for experiments that included N addition, warming, or increasing precipitation because of data limitations for other factors experiments in China.

### Analyses

We used meta-analysis to evaluate how the main processes and properties related to ecosystem carbon and nitrogen cycles respond to major global change drivers^46^,^47^,^48^, and summarized the distribution and characteristics of experiments in China. We calculated response ratios (RR) for each selected variable and experiment^49^. We estimated the effect size of global change factors for each individual observation using the natural log of the response ratio. RR is calculated as the ratio of the mean value of a given variable in the treatment group (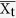) to that in the control group (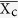) (Eq. 1).


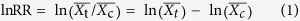


The logarithm of RR is a suitable measure for meta-analyses as its bias is small and its sampling distribution is approximately normal^18^,^47^,^49^. Its variance (*v*) is calculated by


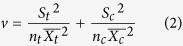


where *n*_*t*_ and *n*_*c*_ are the sample sizes of the concerned variable in treatment and control groups, respectively; *S*_*t*_ and *S*_*c*_ are the standard deviations for the treatment and control groups, respectively. The reciprocal of its variance was used as the weighting factor (*w*_*ij*_) for each In RR (Eq. 3).


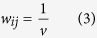


The weighted response ratio (*RR*_++_) and confidence interval (CI) was calculated using the meta-analytical software, METAWIN 2.1 (Sinauer Associates, Inc. Sunderland, MA, USA). Using the mixed-effects model of METAWIN 2.1, the *RR*_++_ was calculated from RR values of individual pairwise comparisons between treatment and control, *RR*_*ij*_ (i = 1, 2,…, m; j = 1, 2,…, k) as in Eq. 4. Here, m is the number of groups (e.g. ecosystem types), and k is the number of comparisons in the *i*th group.


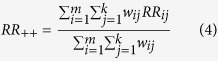


The standard error of *RR*_++_ and 95%CI was estimated by Eq. 5 and Eq. 6, respectively.


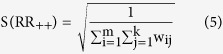



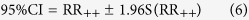


The effect on a response variable was considered significant if the 95% CI did not overlap zero^15^,^36^.

## Additional Information

**How to cite this article**: Fu, Z. *et al.* What have we learned from global change manipulative experiments in China? A meta-analysis. *Sci. Rep.* **5**, 12344; doi: 10.1038/srep12344 (2015).

## Supplementary Material

Supplementary Materials

Supplementary Database

## Figures and Tables

**Table 1:** Abbreviations of ecosystem carbon and nitrogen variables.

Abbreviations	Full name
AGB	Aboveground biomass
BGB	Belowground biomass
LCP	Litter carbon pool
MBC	Microbial biomass carbon
DOC	Soil dissolved organic carbon
SCP	Soil carbon pool
Rs	Soil respiration
ER	Ecosystem respiration
NEP (NEE)	Net ecosystem production (exchange)
GEP	Gross ecosystem production
Rm	Microbial respiration
NPR	Net photosynthetic rate
R/S	Root : shoot ratio
Soil C/N	Soil carbon : nitrogen ratio
Total N	Soil total nitrogen concentration
SIN	Soil inorganic nitrogen
NH_4_^+^-N	Soil NH_4_^+^-N concentration
NO_3_^−^-N	Soil NO_3_^−^-N concentration
MBN	Microbial biomass nitrogen
