# Supplementary material for: What have we learned from global change manipulative experiments in China? A meta-analysis
Source: Sci Rep. 2015 Jul 24;5:12344. doi: 10.1038/srep12344 (PMC4513293; doi:10.1038/srep12344)
Supplement: Supplementary Materials [file srep12344-s1.doc]

**Supplementary materials**

**What have we learned from global change manipulative experiments in China? A meta-analysis**

Zheng Fu^1,2^, Shuli Niu^1^*, Jeffrey S. Dukes^3^

^1^Key Laboratory of Ecosystem Network Observation and Modeling, Institute of Geographic Sciences and Natural Resources Research, CAS, Beijing 100101, China

^2^University of Chinese Academy of Sciences, No.19A Yuquan Road, Beijing, 100049, China

^3^Department of Forestry and Natural Resources, and Department of Biological Sciences, Purdue University, West Lafayette, IN, USA

**Table S1**

**Site characteristics for global change manipulation experiments in China.**

| **Site location** | **Ecosystem types** | **Experiments** | **Reference** |
| --- | --- | --- | --- |
| 18°43´N, 108°53´E | Tropical montane rain forest | N addition;  P addition | Du et al. 2013 |
| 21°56'N, 101°16'E | Tropical rain forest | Precipitation changing | Dong et al. 2012 |
| 23°08'N, 112°35'E | Subtropical forest | N addition;  Precipitation changing | Wang et al.2008;  Fang et al.2012 |
| 23°10'N, 112°10'E | Subtropical forest | N addition;  P addition;  Precipitation changing | Chen et al.2012;  Chen et al.2013;  Fang et al.2006;  Liu et al.2013;  Lu et al.2009;  Lu et al.2010;  Lu et al.2012;  Mo et al.2006, 2007, 2008;  Zhang et al.2008;  Jiang et al.2013 |
| 23°10'N, 113°33'E | Subtropical forest | N addition | Zhu et al.2013 |
| 23°19´N, 112°19´E | Subtropical forest | N addition | Fang et al.2007 |
| 23°20'N, 113°30' E | Subtropical forest | N addition;  P addition;  Precipitation changing;  Elevated CO_2_ | Duan et al.2009;  Deng et al.2010;  Chen et al.2012;  Liu et al.2010 |
| 23°39'N, 113°17'E | Subtropical forest | N addition | Cai et al.2013 |
| 24°32' N, 101°01' E | Subtropical forest | Warming | Zhang et al. 2015 |
| 26°19'N, 117°36'E | Forest | Warming | Chen, et al. 2013 |
| 26°30´N, 117°43´E | Forest | N addition | Fan et al.2007;  Wu et al.2013 |
| 26°31´N, 109°47´E | Forest | N addition | Wang et al.2008 |
| 27°03'N, 118°09'E | Forest | N addition | Yan et al.2012 |
| 27°39‘N, 117°57‘E | Subtropical evergreen broadleaved forest | N addition | Du, et al. 2013 |
| 28°02´-06´N, 113°02-07´E | Forest | N addition | Zhang et al.2012;  Zhang et al.2012;  Zhen et al.2013 |
| 28°09–29°11'N, 115°27'–116°35'E | Forest | N addition;  P addition | Chen et al.2012 |
| 28°29-54´N, 103°47-49´E | Evergreen broadleaved forest | N addition | Song et al.2007 |
| 29°10´-17´N, 118°45´-122°E | Forest | N addition | Wu et al.2011 |
| 29°95' N, 103°38'E | Forest | N addition | Tu et al.2011 |
| 29°20'-30º20'N, 101º30'-102º15' E | Forest | Warming | Yang et al.2011;  Yang et al.2013 |
| 30°01‘N, 117°21‘E | Subtropical evergreen broadleaved forest | N addition | Du, et al. 2013 |
| 30°08'N, 103°00'E | Forest | N addition | Li et al.2010 |
| 30°14'N, 119°42'E | Forest | N addition | Li et al.2011 |
| 30°30’N, 91°03’E | Grassland | Warming | Fu et al.2012;  Zhao et al.2010 |
| 30°51' N, 91°05' E | Grassland | N addition | Zong et al.2013;  Jiang et al.2013 |
| 31°35´N, 102°50´E | Forest | Warming | Pan et al.2008;  Xu et al.2010;  Yin et al.2008;  Han et al.2009 |
| 31°41´N, 103°51´E | Forest | N addition | Liu et al.2011;  Chen et al.2010;  Zhao et al.2009 |
| 31°41'N, 103°53'E | Subalpine forests | Warming;  Elevated CO_2_ | Zhao et al.2009;  Yin et al.2012;  Xiong et al.2010;  Chen et al.2010;  Feng et al.2007;  Qiao et al.2007;  Yang et al.2010;  Liu et al.2011 |
| 31°46'N, 114°05'E | Deciduous broadleaved mixed forest | N addition;  P addition;  Precipitation changing | http://hs.scib.ac.cn/inc/hsf_platform/jigongshan.html |
| 31°50´-33°22´N, 101°51´-103°23´E | Grassland | Warming | Li et al.2011 |
| 32°03'N, 118°51'E | Forest | N addition | Li et al.2010 |
| 32°11'N, 118°42'E | Forest | N addition | Li et al.2010 |
| 32°27´N, 102°22´E | Grassland | Warming | Wang et al.2011 |
| 32°51´N, 103°33´E | Grassland | Warming | Shi et al.2008 |
| 32°59'N, 104°01'E | Subalpine forests | Warming | Xu et al.2010 |
| 32°93'N, 104°08'E | Grassland | Warming | Xu et al.2009 |
| 34°17-25’N, 100°26-43’E | Grassland | Warming | Liu et al.2010 |
| 34°43’N, 92°53’E | Grassland | Warming | Li et al.2011 |
| 36°18'-37°05'N, 111°45'-112°33'E | Forest | N addition | Guo et al.2013 |
| 36°31-43'N, 112°01-15'E | Forest | N addition | Li et al.2014 |
| 37°25´N, 104°35´E | Grassland | N addition | Su et al.2012 |
| 37°30´N, 101°20´E | Grassland | Warming | Li et al.2004;  Zhou et al.2000;  Jing et al.2013 |
| 37°37´N, 101°12´E | Grassland | Warming;  N addition;  Precipitation changing;  Clipping | Klein et al.2007;  Lin et al.2011;  Luo et al.2009;  Rui et al.2011;  Wang et al.2012;  Shen et al.2002;  Zhu et al.2011 |
| 37°58'N, 100°55'E | Grassland | Warming;  Precipitation changing | Heng et al.2010 |
| 38° 34'N, 102° 58' E | Desert | Precipitation changing | Song et al.2012 |
| 39°21'N, 124°30'E | Forest | N addition | Yu et al.2007 |
| 41°46 N, 111°53′ E | Grassland | Warming | Wang et al.2012 |
| 42°01'N, 128°06'E | Forest | Precipitation changing | Chen et al.2011 |
| 42°02´N, 116°17´E | Grassland | Warming;  N addition;  P addition;  Precipitation changing;  Elevated CO_2_;  Clipping | Li et al.2013;  Liu et al.2009;  Niu et al.2007;  Xia et al.2009;  Xia et al.2012;  Yang et al.2010;  Chi et al.2013;  Bi et al.2012;  Xiao et al.2007 |
| 42°24' N, 128°28' E | Forest | Warming;  N addition;  Precipitation changing;  Elevated CO_2_ | Wang et al.2003;  Wang et al.2004;  Hu et al.2009;  Fan et al.2010 |
| 42°25'N, 117°15'E | Forest | N addition | Du, et al. 2013;  Wei et al.2008 |
| 42°45´N, 116°67´E | Grassland | N addition | Xu et al.2008 |
| 42°58´N, 122°21´E | Grassland | N addition | Li et al.2009 |
| 43°26´-44°N, 115°32´-117°E | Grassland | Precipitation changing | Chen et al.2008 |
| 43°32´-33'N, 116°40´E | Grassland | N addition | Pan et al.2005 |
| 43°38'N, 116°42' E | Grassland | N addition | Zhang et al.2009;  Bai et al.2009 |
| 44.17° N, 87.56° E | Desert | N addition;  Precipitation changing | Zhou et al.2010 |
| 44°22'N, 87°55'E | Desert | Precipitation changing | Zhao et al.2014 |
| 44°45'N, 123°45'E | Grassland | Warming;  N addition | Qi et al.2012 |
| 45°21´N, 127°30´E | Forest | N addition | Jia et al.2007;  Jia et al.2009;  Zhao et al.2010 |
| 48°07'N, 129°11'E | Forest | N addition | Du, et al. 2013 |
| 48°02-12'N, 128°58'-129°15' E | Forest | N addition | Liu et al.2012 |
| 49° 19'N, 120° 02' E | Grassland | Precipitation changing | Ma et al.2012 |
| 50°20´-30´N, 121°45´-122°E | Forest | N addition | Wen et al.2012;  Gao et al.2013 |
| 50°56'N, 121°30'E | Forest | N addition | Du, et al. 2013 |


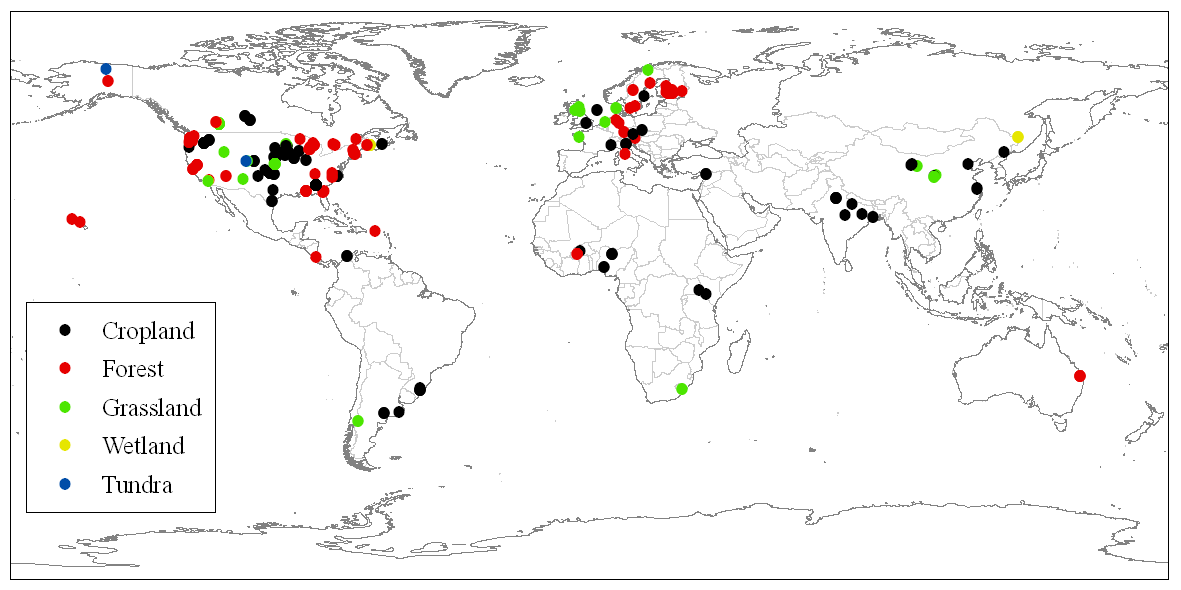


**Fig. S1** The distribution of N addition experiments at global scale (Lu et al. 2011). The red area stands for the latitudinal range of China. The figure shows that lots of N addition experiments conducted in China missed in the global synthesis.


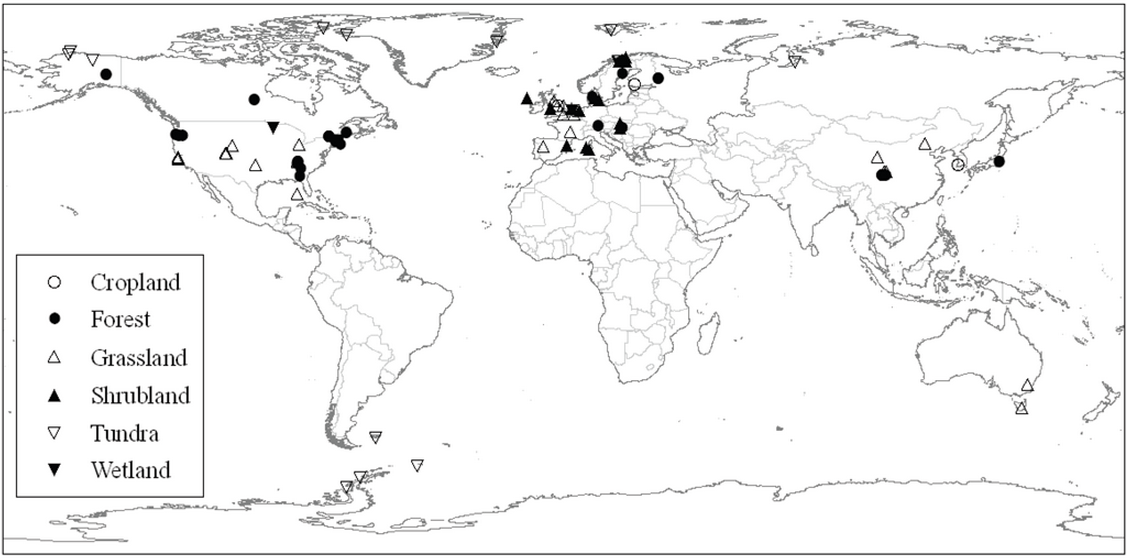
**Fig. S2** The distribution of warming experiments at global scale (Lu et al. 2013). The red area stands for the latitudinal range of China. The figure shows that lots of warming experiments conducted in China missed in the global synthesis.

**References**

Bai W, Wan S, Niu S*, et al.* 2010. Increased temperature and precipitation interact to affect root production, mortality, and turnover in a temperate steppe: Implications for ecosystem c cycling Global Change Biol **16**: 1306-1316.

Bai Y, Wu J, Clark CM*, et al.* 2010. Tradeoffs and thresholds in the effects of nitrogen addition on biodiversity and ecosystem functioning: Evidence from inner mongolia grasslands Global Change Biol **16**: 358-372.

Bi J, Zhang N, liang Y*, et al.* 2012. Impacts of increased n use and precipitation on microbial c utilization potential in the semiarid grassland of inner mongolia（in chinese with english abstract） Chinese Journal of Eco-Agriculture **20**: 1586-1593.

Cai Y, Huang Y, Zhang T*, et al.* 2013. Effects of simulated nitrogen deposition on the vertical distribution of soil nitrogen, carbon and microbial biomass in young schima superb plantation（in chinese with english abstract） Ecology and Environmental Sciences **5**: 006.

Chen H, Dong S, Liu L*, et al.* 2013. Effects of experimental nitrogen and phosphorus addition on litter decomposition in an old-growth tropical forest Plos One **8**: e84101.

Chen S, Lin G, Huang J*, et al.* 2008. Responses of soil respiration to simulated precipitation pulses in semiarid steppe under different grazing regimes J Plant Ecol **1**: 237-246.

Chen S, Lin G, Huang J*, et al.* 2009. Dependence of carbon sequestration on the differential responses of ecosystem photosynthesis and respiration to rain pulses in a semiarid steppe Global Change Biol **15**: 2450-2461.

Chen X, Li Y, Mo J*, et al.* 2012. Effects of nitrogen deposition on soil organic carbon fractions in the subtropical forest ecosystems of s china J Plant Nutr Soil Sc **175**: 947-953.

Chen X, Liu J, Deng Q*, et al.* 2012. Effects of elevated co2 and nitrogen addition on soil organic carbon fractions in a subtropical forest Plant Soil **357**: 25-34.

Chen Z, Yin H, Wei Y*, et al.* 2010. Short-term effects of night warming and nitrogen addition on soil available nitrogen and microbial properties in subalpine coniferous forest, western sichuan, china（in chinese with english abstract） Chinese Journal of Plant Ecology:

Chi Y, Xu M, Shen R*, et al.* 2013. Acclimation of foliar respiration and photosynthesis in response to experimental warming in a temperate steppe in northern china Plos One **8**: e56482.

Deng Q, Hui D, Zhang D*, et al.* 2012. Effects of precipitation increase on soil respiration: A three-year field experiment in subtropical forests in china Plos One **7**: e41493.

Deng Q, Zhou G, Liu J*, et al.* 2009. Effects of co_2_ enrichment, high nitrogen deposition and high precipitation on a model forest ecosystem in southern china（in chinese with english abstract） Chinese Journal of Plant Ecology **33**: 1023-1033.

Deng Q, Zhou G, Liu J*, et al.* 2010. Responses of soil respiration to elevated carbon dioxide and nitrogen addition in young subtropical forest ecosystems in china Biogeosciences **7**: 315-328.

Duan H, Liu J, Deng Q*, et al.* 2009. Effects of elevated co2 and n deposition on plant biomass accumulation and allocation in subtropical forest ecosystems: A mesocosm study（in chinese with english abstract） Chinese Journal of Plant Ecology **33**: 570-579.

Fan H, Liu W, Li Y*, et al.* 2007. Tree growth and soil nutrients in response to nitrogen deposition in a subtropical chinese fir plantation（in chinese with english abstract） ACTA ECOLOGICA SINICA **11**:

Fan H, Liu W, Yan Y*, et al.* 2008. Decomposition of leaf litter of chinese fir in response to increased nitrogen deposition（in chinese with english abstract） Journal of Beijing Forestry University **30**: 8-13.

Fang H, Mo J, Peng S*, et al.* 2007. Cumulative effects of nitrogen additions on litter decomposition in three tropical forests in southern china Plant Soil **297**: 233-242.

Fang X, Liu J, Zhang D*, et al.* 2012. Effects of precipitation change and nitrogen addition on organic carbon mineralization and soil microbial carbon of the forest soils in dinghushan,southeastern china（in chinese with english abstract） Chin J Appl Environ Biol **18**: 531-538.

Fang Y-t, Zhu W-x, Mo J-m*, et al.* 2006. Dynamics of soil inorganic nitrogen and their responses to nitrogen additions in three subtropical forests, south china Journal of Environmental Sciences **18**: 752-759.

Fu G, Shen Z, Zhang X*, et al.* 2012. Response of soil microbial biomass to short-term experimental warming in alpine meadow on the tibetan plateau Appl Soil Ecol **61**: 158-160.

Fu G, Zhang X, Zhang Y*, et al.* 2013. Experimental warming does not enhance gross primary production and above-ground biomass in the alpine meadow of tibet J Appl Remote Sens **7**: 073505.

Gao W, Chen S, Fang H*, et al.* 2013. Early responses of soil ch_4_ uptake to increased atmospheric nitrogen deposition in a cold-temperate coniferous forest（in chinese with english abstract） Acta Ecologica Sinica **33**: 7505-7515.

Guo Y, Fan X, Wang J*, et al.* 2013. Responses of soil microbial biomass to simulated nitrogen deposition in pinustabulaeformisforests in the taiyue mountain of china Chinese Journal of Appplied Environmental Biology **19**: 605-610.

Han C, Liu Q and Yang Y. 2009. Short-term effects of experimental warming and enhanced ultraviolet-b radiation on photosynthesis and antioxidant defense of picea asperata seedlings Plant Growth Regulation **58**: 153-162.

Heng T, Wu J, Xie S*, et al.* 2011. The responses of soil c and n, microbial biomass c or n under alpine meadow of qinghai-tibet plateau to the change of temperature and precipitation（in chinese with english abstract） Chinese Agricultural Science Bulletin **27**: 425-430.

Hu Y, Han S, Li X*, et al.* 2009. Responses of soil available nitrogen of natural forest and secondary forest to simulated n deposition in changbai mountain（in chinese with english abstract） Journal of Northeast Forestry University **37**: 36-38.

Hu Z, Li H, Yang Y*, et al.* 2010. Effect of simulated nitrogen deposition on soil respiration in northern subtropical deciduous broad-leave forest（in chinese with english abstract） EVIRONMENTAL SCIENCE **31**: 1726-1732.

Jia S, Wang Z, Mei L*, et al.* 2007. Effect of nitrogen fertilization on soil respiration in larix gmelinii and fraxinus mandshurica plantations in china（in chinese with english abstract） Chinese Journal of Plant Ecology **31**: 372-379.

Jia S, Zhao Y, Sun Y*, et al.* 2009. Effects of nitrogen fertilization on seasonal dynamics of soil microbial biomass carbon and nitrogen in larix gmelinii and fraxinus mandshurica plantations Chinese Journal of Applied Ecology: 2063-2071.

Jiang H, Deng Q, Zhou G*, et al.* 2013. Responses of soil respiration and its temperature/moisture sensitivity to precipitation in three subtropical forests in southern china Biogeosciences **10**: 3963-3982.

Jiang J, Zong N, Song M*, et al.* 2013. Responses of ecosystem respiration and its components to fertilization in an alpine meadow on the tibetan plateau Eur J Soil Biol **56**: 101-106.

Jing X, Wang Y, Chung H*, et al.* 2013. No temperature acclimation of soil extracellular enzymes to experimental warming in an alpine grassland ecosystem on the tibetan plateau Biogeochemistry **117**: 39-54.

Klein JA, Harte J and Zhao X-Q. 2007. Experimental warming, not grazing, decreases rangeland quality on the tibetan plateau Ecol Appl **17**: 541-557.

Li G, Liu Y, Frelich LE*, et al.* 2011. Experimental warming induces degradation of a tibetan alpine meadow through trophic interactions J Appl Ecol **48**: 659-667.

Li H, Wang J, Liu X*, et al.* 2014. Effects and its sustained effect of simulated nitrogen deposition on soil respiration in pinus tabulaeformis forests in the taiyue mountain, china（in chinese with english abstract） Acta Scientiae Circumstantiae:

Li K, Jiang H, You M*, et al.* 2011. Effect of simulated nitrogen deposition on the soil respiration of *lithocarpus glabra* and *castanopsis sclerophylla*（in chinese with english abstract） Acta Ecologica Sinica **31**: 82-89.

Li L, Zeng D, Yu Z*, et al.* 2009. Effects of nitrogen addition on grassland species diversity and productiv ity in keerqin sandy land（in chinese with english abstract） Chinese Journal of Applied Ecology **20**: 1838-1844.

Li N, Wang G, Gao Y*, et al.* 2011. Warming effects on plant growth, soil nutrients, microbial biomass and soil enzymes activities of two alpine meadows in tibetan plateau Pol J Ecol **59**: 25-35.

Li Q, Bai H, Liang W*, et al.* 2013. Nitrogen addition and warming independently influence the belowground micro-food web in a temperate steppe Plos One **8**: e60441.

Li R, Tu L, Hu T*, et al.* 2010. Effects of simulated nitrogen deposition on soil respiration in a *neosinocalamus affinis* plantation in rainy area of west china（in chinese with english abstract） Chinese Journal of Applied Ecology: 1649-1655.

Li Y, Zhao L, Zhao X*, et al.* 2004. Effects of a 5 year mimic temperature increase to structure and productivity of *kobresia humilis* meadow（in chinese with english abstract） ACTA AGRESTIA SINICA **12**: 236-239.

Lin X, Zhang Z, Wang S*, et al.* 2011. Response of ecosystem respiration to warming and grazing during the growing seasons in the alpine meadow on the tibetan plateau Agr Forest Meteorol **151**: 792-802.

Liu B, Mu C, Xing Y*, et al.* 2013. Efects of simulated nitrogen deposition on soil respiration in spruce-fir-korean pine forest of xiaoxing'anling mountains china（in chinese with english abstract） Forest Research **25**: 767-772.

Liu F, Wang H and Zhang Y. 2013. Effects of elevated temperature and doubled co_2_ concentration on soil dissolved organic carbon and nitrogen in a subalpine coniferous forest of western sichuan,southwest china（in chinese with english abstract） Chinese Journal of Ecology **32**: 2844-2849.

Liu JX, Zhou GY, Zhang DQ*, et al.* 2010. Carbon dynamics in subtropical forest soil: Effects of atmospheric carbon dioxide enrichment and nitrogen addition J Soil Sediment **10**: 730-738.

Liu L, Zhang T, Gilliam FS*, et al.* 2013. Interactive effects of nitrogen and phosphorus on soil microbial communities in a tropical forest Plos One **8**: e61188.

Liu Q, Yin H, Chen J*, et al.* 2011. Belowground responses of picea asperata seedlings to warming and nitrogen fertilization in the eastern tibetan plateau Ecol Res **26**: 637-648.

Liu W, Zhang ZHE and Wan S. 2009. Predominant role of water in regulating soil and microbial respiration and their responses to climate change in a semiarid grassland Global Change Biol **15**: 184-195.

Liu Wei, Wang Changting, Zhao Jianzhong*, et al.* 2010. Responses of quantity characteristics of plant community to simulating warming in alpine *kobresia humilis* meadow ecosystem（in chinese with english abstract）Acta Bot Boreal-Occident Sin **30**: 0995-1003.

Lu, M., Y. Yang, Y. Luo, C. Fang, X. Zhou, J. Chen, X. Yang, and B. Li. 2011a. Responses of ecosystem nitrogen cycle to nitrogen addition: a meta-analysis. New Phytologist **189**:1040-1050.

Lu, M., X. Zhou, Q. Yang, H. Li, Y. Luo, C. Fang, J. Chen, X. Yang, and B. Li. 2013. Responses of ecosystem carbon cycle to experimental warming: a meta-analysis. Ecology **94**:726-738.

Lu X, Mo J, Gilliam FS*, et al.* 2012. Nitrogen addition shapes soil phosphorus availability in two reforested tropical forests in southern china Biotropica **44**: 302-311.

Lu X, Mo J, Gilliam FS*, et al.* 2010. Effects of experimental nitrogen additions on plant diversity in an old-growth tropical forest Global Change Biol **16**: 2688-2700.

Lu X-K, Mo J-M, Gundersern P*, et al.* 2009. Effect of simulated n deposition on soil exchangeable cations in three forest types of subtropical china Pedosphere **19**: 189-198.

Luo C, Xu G, Chao Z*, et al.* 2010. Effect of warming and grazing on litter mass loss and temperature sensitivity of litter and dung mass loss on the tibetan plateau Global Change Biol **16**: 1606-1617.

Luo C, Xu G, Wang Y*, et al.* 2009. Effects of grazing and experimental warming on doc concentrations in the soil solution on the qinghai-tibet plateau Soil Biology and Biochemistry **41**: 2493-2500.

Luo Y, Sherry R, Zhou X*, et al.* 2009. Terrestrial carbon-cycle feedback to climate warming: Experimental evidence on plant regulation and impacts of biofuel feedstock harvest GCB Bioenergy **1**: 62-74.

Mo J, Brown S, Xue J*, et al.* 2006. Response of litter decomposition to simulated n deposition in disturbed, rehabilitated and mature forests in subtropical china Plant Soil **282**: 135-151.

Mo J, Fang Y, Xu G*, et al.* 2005. The short-term responses of soil co2 emission and ch4 uptake to simulated n deposition in nursery and forests of dinghushan in subtropical china（in chinese with english abstract） ACTA ECOLOGICA SINICA **25**: 682-690.

Mo J, Li D and Gundersen P. 2008. Seedling growth response of two tropical tree species to nitrogen deposition in southern china Eur J Forest Res **127**: 275-283.

Mo J, Zhang W, Zhu W*, et al.* 2007. Response of soil respiration to simulated n deposition in a disturbed and a rehabilitated tropical forest in southern china Plant Soil **296**: 125-135.

Mo J, Zhang WEI, Zhu W*, et al.* 2007. Nitrogen addition reduces soil respiration in a mature tropical forest in southern china Global Change Biol **14**: 403-412.

Na L, Genxu W, Yan Y*, et al.* 2011. Plant production, and carbon and nitrogen source pools, are strongly intensified by experimental warming in alpine ecosystems in the qinghai-tibet plateau Soil Biology and Biochemistry **43**: 942-953.

Niu S, Wu M, Han Y*, et al.* 2008. Water-mediated responses of ecosystem carbon fluxes to climatic change in a temperate steppe New Phytol **177**: 209-19.

Niu S, Wu M, Han Y*, et al.* 2010. Nitrogen effects on net ecosystem carbon exchange in a temperate steppe Global Change Biol **16**: 144-155.

Niu S, Yang H, Zhang Z*, et al.* 2009. Non-additive effects of water and nitrogen addition on ecosystem carbon exchange in a temperate steppe Ecosystems **12**: 915-926.

Pan Q, Bai Y, Han X*, et al.* 2005. Effects of nitrogen additions on a leymus chinensis population in typical steppe of inner mongolia（in chinese with english abstract） chinese Journal of Plant Ecology **29**: 311-317.

Pan X, Lin B and Liu Q. 2008. Effects of elevated temperature on soil organic carbon and soil respiration under subalpine coniferous forest in western sichuan province, china（in chinese with english abstract） Chinese Journal of Applied Ecology:

Qi H, Liu Y and Guo J. 2013. Decomposition of *leymus chinensis* and *phragmites communis* litter responses to simulated warming and nitrogen application in songnen grassland（in chinese with english abstract） Journal of Northeast Normal University **44**: 114-119.

Rui Y, Wang S, Xu Z*, et al.* 2011. Warming and grazing affect soil labile carbon and nitrogen pools differently in an alpine meadow of the qinghai–tibet plateau in china J Soil Sediment **11**: 903-914.

Shen Z, Zhou X, Chen Z*, et al.* 2002. Response of plant groups to simulated rainfall and nitrogen supply in alpine kobresia humilis meadow（in chinese with english abstract） Acta Phytoecologica Sinica **26**: 288-294.

Shi F, Chen H and Wu N. 2008. Effect of experimental warming on carbon and n itrogen content of subalpine meadow in northwestern sichuan（in chinese with english abstract） BULLETIN OF BOTAN ICAL RESEARCH:

Shi F, Wu N and Luo P. 2008. Effect of temperature enhancement on community structure and biomass of subalpine meadow in northwestern sichuan（in chinese with english abstract） ACTA ECOLOGICA SIN ICA:

Shi F, Wu N and Wu Y. 2010. Responses of plant growth and substance allocation of three dominant plant species to experimental warming in an alpine grassland, northwestern sichuan, china（in chinese with english abstract） Chinese Journal of Plant Ecology:

Shi F, Wu N, Wu Y*, et al.* 2010. Effect of simulated temperature enhancement on growth and photosynthesis of <i>deschampsia caespitosa</i> and <i>thlaspi arvense</i> in northwestern sichuan, china* Chinese Journal of Appplied Environmental Biology **2009**: 750-755.

Song X, Hu T, Xian J*, et al.* 2007. Responses of litter decomposition and nutrient release to simulated nitrogen deposition in an evergreen broad-leaved forest in southwestern sichuan（in chinese with english abstract） Chinese Journal of Applied Ecology:

Su J, Li X, Li X*, et al.* 2013. Effects of additional n on herbaceous species of desertified steppe in arid regions of china: A four-year field study Ecol Res **28**: 21-28.

Tang X, Liu S, Zhou G*, et al.* 2006. Soil-atmospheric exchange of co2, ch4, and n2o in three subtropical forest ecosystems in southern china Global Change Biol **12**: 546-560.

Tu L, Dai H, Hu T*, et al.* 2011. Effects of simulated nitrogen deposition on soil respiration in a *bambusa pervariabilis* × *dendrocala mopsi* plantation in rainy area of west china（in chinese with english abstract） Chinese Journal of Applied Ecology **22**: 829-836.

Tu L, Hu T, Huang L*, et al.* 2009. Response of soil respiration to simulated nitrogen deposition in pleioblastus amarus forest, rainy area of west china（in chinese with english abstract） Chinese Journal of Plant Ecology **33**: 728-738.

Tu L-H, Hu T-X, Zhang J*, et al.* 2010. Short-term simulated nitrogen deposition increases carbon sequestration in a pleioblastus amarus plantation Plant Soil **340**: 383-396.

Wang B, Sun G, Luo.P.*, et al.* 2011. Labile and recalcitrant carbon and nitrogen pools of an alpine meadow soil from the eastern qinghai-tibetan plateau subjected to experimental warming and grazing（in chinese with english abstract） Acta Ecologica Sinica:

Wang H, Mo J, Lu X*, et al.* 2008. Effects of elevated nitrogen deposition on soil microbial biomass carbon in the main subtropicalf orests of southern china（in chinese with english abstract） ACTA ECOLOGICA SINICA **28**: 470-478.

Wang H, Zhou G-S, Jiang Y-L*, et al.* 2013. Interactive effects of changing precipitation and elevated co<sub>2</sub> concentration on photosyn-thetic parameters of <i>stipa breviflora</i> Chinese Journal of Plant Ecology **36**: 597-606.

Wang QK, Wang SL and Liu YX. 2008. Responses to n and p fertilization in a young eucalyptus dunnii plantation: Microbial properties, enzyme activities and dissolved organic matter Appl Soil Ecol **40**: 484-490.

Wang S, Duan J, Xu G*, et al.* 2012. Effects of warming and grazing on soil n availability, species composition, and anpp in an alpine meadow Ecology **93**: 2365-2376.

Wang Z, Zhao M, Han G*, et al.* 2012. Response of soil respiration to simulated warming and n addition in the desert steppe（in chinese with english abstract） Journal of Arid Land Resources and Environment **26**: 98-103.

Wei T, Ren Y, Zeng H*, et al.* 2009. Effects of throughfall manipulation on the dynamics of soil microbial biomass carbon and microbial quotient in a *pinus sylvetris* var. *Mongolica* plantation（in chinese with english abstract） Acta Scientiarum Naturalium Universitatis Pekinensis **45**: 533-540.

Wu J, Liu W, Fan H*, et al.* 2013. Asynchronous responses of soil microbial community and understory plant community to simulated nitrogen deposition in a subtropical forest Ecol Evol **3**: 3895-905.

Wu Q, Ding J, Yan H*, et al.* 2011. Effects of simulated precipitation and nitrogen addition on seedling growth and biomass in five tree species in gutian mountain, zhejiang province, china（in chinese with english abstract） Chinese Journal of Plant Ecology **35**: 256-267.

Xia J, Niu S and Wan S. 2009. Response of ecosystem carbon exchange to warming and nitrogen addition during two hydrologically contrasting growing seasons in a temperate steppe Global Change Biol **15**: 1544-1556.

Xia J and Wan S. 2012. The effects of warming-shifted plant phenology on ecosystem carbon exchange are regulated by precipitation in a semi-arid grassland Plos One **7**: e32088.

Xiao C, Janssens IA, Liu P*, et al.* 2007. Irrigation and enhanced soil carbon input effects on below-ground carbon cycling in semiarid temperate grasslands New Phytol **174**: 835-46.

Xiong P, Xu Z, Lin B, Liu Q (2010) Short-term response of winter soil respiration to simulated warming in a *Pinus armandii plantation* in the upper reaches of the Minjiang River, China (in Chinese with English abstract). *Chinese Journal of Plant Ecology,* **34**.

Xu W and Wan S. 2008. Water-and plant-mediated responses of soil respiration to topography, fire, and nitrogen fertilization in a semiarid grassland in northern china Soil Biology and Biochemistry **40**: 679-687.

Xu Z, Hu T, Li X*, et al.* 2009. Short-term responses of grass community in clear-cutting land of sub--alpine regions to simulated global warming,western sichuan（in chinese with english abstract） ACTA ECOLOGICA SINICA:

Xu Z, Hu T, Zhang L*, et al.* 2010. Short-term gas exchange responses of betula utilis to simulated global warming in a timberline ecotone, eastern tibetan plateau, china（in chinese with english abstract） Chinese Journal of Plant Ecology:

Xu Z-f, Hu R, Xiong P*, et al.* 2010. Initial soil responses to experimental warming in two contrasting forest ecosystems, eastern tibetan plateau, china: Nutrient availabilities, microbial properties and enzyme activities Appl Soil Ecol **46**: 291-299.

Yan L, Chen S, Huang J*, et al.* 2011. Water regulated effects of photosynthetic substrate supply on soil respiration in a semiarid steppe Global Change Biol **17**: 1990-2001.

Yang B, Wang J and Zhang Y. 2010. Effect of long-term warming on growth and biomass allocation of *abies faxoniana* seedlings（in chinese with english abstract） ACTA ECOLOGICA SINICA:

Yang H, Wu M, Liu W*, et al.* 2011. Community structure and composition in response to climate change in a temperate steppe Global Change Biol **17**: 452-465.

Yang L, Yang Y, Wang G*, et al.* 2011. Short-term effects of warming on growth and stoichiometrical characteristics of *abies fabiri* (mast.) craib seedling in gongga mountain（in chinese with english abstract） ACTA ECOLOGICA SINICA **31**: 3668-3676.

Yin H, Chen Z and Liu Q. 2012. Effects of experimental warming on soil n transformations of two coniferous species, eastern tibetan plateau, china Soil Biology and Biochemistry **50**: 77-84.

Yin HJ, Liu Q and Lai T. 2007. Warming effects on growth and physiology in the seedlings of the two conifers picea asperata and abies faxoniana under two contrasting light conditions Ecol Res **23**: 459-469.

Yu L, Ding G, Zhu J*, et al.* 2007. Effects of fertiliza tion on f ine root b ioma ss of l a rix kaem pferi plan ta tion（in chinese with english abstract） Chinese Journal of App lied Ecology **18**: 713-720.

Zeng D-H, Li L-J, Fahey TJ*, et al.* 2010. Effects of nitrogen addition on vegetation and ecosystem carbon in a semi-arid grassland Biogeochemistry **98**: 185-193.

Zhang L, Huang J, Bai Y*, et al.* 2009. Effects of nitrogen addition on net nitrogen mineralization in *leymus chinensis* grassland, inner mongolia, china（in chinese with english abstract） Chinese Journal of Plant Ecology **33**: 563-569.

Zhang T, Zhu W, Mo J*, et al.* 2011. Increased phosphorus availability mitigates the inhibition of nitrogen deposition on ch 4 uptake in an old-growth tropical forest, southern china Biogeosciences **8**: 2805-2813.

Zhang W, Mo J, Yu G*, et al.* 2008. Emissions of nitrous oxide from three tropical forests in southern china in response to simulated nitrogen deposition Plant Soil **306**: 221-236.

Zhang X, Yan W, Ma X*, et al.* 2012. Short-term effects of nitrogen deposition on soil respiration of *cinnamomum camphora* plantation（in chinese with english abstract） Journal of Central South University of Forestry & Technology **32**: 109-113.

Zhang X, Yan W, Zhen W*, et al.* 2012. Effects of nitrogen deposition on soil respiration of *pinus elliottii*（in chinese with english abstract） Chinese Agricultural Science Bulletin **28**: 5-10.

Zhao C and Liu Q. 2008. Growth and photosynthetic responses of two coniferous species to experimental warming and nitrogen fertilization Canadian Journal of Forest Research **39**: 1-11.

Zhao Q, Liu X, Hu Y*, et al.* 2010. Effects of nitrogen addition on nutrient allocation and nutrient resorption efficiency in *larix gmelinii*（in chinese with english abstract） SCIENTIA SILVAE SINICAE **46**: 14-19.

Zhen W, Yan W, Wang G*, et al.* 2013. Effect of nitrogen addition to soil respiration in *cinnamomum camphora* forest in subtropical china（in chinese with english abstract） ACTA ECOLOGICA SINICA **33**: 3425-3433.

Zhou H, Zhou X, Zhao X*, et al.* 2000. A preliminary study of the influence of simulated greenhouse effect on a kobresia humilis meadow（in chinese with english abstract） Chinese Journal of Plant Ecology:

Zhu F, Yoh M, Gilliam FS*, et al.* 2013. Nutrient limitation in three lowland tropical forests in southern china receiving high nitrogen deposition: Insights from fine root responses to nutrient additions Plos One **8**: e82661.

Zhu T, Chen S, Fang H*, et al.* 2011. Early responses of soil co2 emission to simulating atmospheric nitrogen deposition in an alpine meadow on the qinghai tibetan plateau（in chinese with english abstract） ACTA ECOLOGICA SINICA **31**: 2687-2696.

Zong N, Shi P, Jiang J*, et al.* 2013. Interactive effects of short-term nitrogen enrichment and simulated grazing on ecosystem respiration in an alpine meadow on the tibetan plateau（in chinese with english abstract） ACTA ECOLOGICA SINICA **33**: 6191-6201.

Zong N, Shi P, Song M*, et al.* 2012. Clipping alters the response of biomass allocation pattern under nitrogen addition in an alpine meadow on the tibetan plateau（in chinese with english abstract） JOURNAL OF NATURAL RESOURCES: 1696-1707.

**Literatures included in Fig.1 and Table. S1 but not included in the meta-analysis.**

Cong-wei., Y., Hong-liang., M., Ren., G. & Yun-feng., Y. (2012) Effects of Simulated Nitrogen Deposition on Soluble Nitrogen in Subtropical Forest Soils. *Environmental Sciences*, **25**, 678-384.

Du, E., Zhou, Z., Li, P., Hu, X., Ma, Y., Wang, W., Zheng, C., Zhu, J., He, J. & Fang, J. (2013) NEECF: a project of nutrient enrichment experiments in China’s forests. *Journal of Plant Ecology*, rtt008.

Jinjuan., F., Xianjing., M., Xinyu., Z. & Xiaomin, S. (2010) Forest soil organic matter δ^13^ C along a altitudinal transect on northern slope of Changbai Mountains under effects of simulated warming (in Chinese with English abstract). *Chinese Journal of Applied Ecology*, **21**, 1621-1626.

Liyuan., D., Chuan-sheng., W., Jian-mei., G. & Li-qing., S. (2012) Effects of simulated rainfall on the soil respiration in tropical secondary forest and rubber plantation in Xishuangbanna of Yunnan, Southwest China (in Chinese with English abstract). *Chinese Journal of Ecology*, **31**, 1887-1892.

Ma, L., Huang, W., Guo, C., Wang, R. & Xiao, C. (2012) Soil microbial properties and plant growth responses to carbon and water addition in a temperate steppe: the importance of nutrient availability. *PloS one*, **7**, e35165.

Miao, W., Qiu-rong, L., Li-min, D. & Lan-zhu, J. (2003) Response of seedlings of different tree species to elevated CO2 in Changbai Mountain. *Journal of Forestry Research*, **14**, 112-116.

Miao, W., Qiu-rong, L., Zhanqing, H. & Li-min, D. (2004) Effects of soil water regimes on the growth of *Quercus mongolica* seedlings in Changbai Mountains. *CHINESE JOURNAL OF APPLIED ECOLOGY*, **15**, 1765-1770.

Rui-Fang., F., Wan-Qin., Y., Jian., Z. & Ren-Ju., D. (2007) Effects of simulated elevated atmospheric CO2 concentration and temperature on soil enzyme activity in the subalpine fir forest (in Chinese with English abstract). *ACTA ECOLOGICA SINICA*, **27**

Shi-dong., C., Xiao-fei., L., De-cheng., X. & Wei-sheng., L. (2013) A Preliminary Study on Effects of Continuous Active Warming on Soil Respiration Rates in Central Sub-tropical Forests (in Chinese with English abstract). *Journal of Subtropical Resources and Environment*, **8**

Xin-Feng, Z., Hai-Liang, X.U., Peng, Z. & Zhang Qing-Qing, a. (2014) Influence of nutrient and water additions on functional traits of Salsola nitraria in desert grassland (in Chinese with English abstract). *Chinese Journal of Plant Ecology*, **38**, 134-146.

Xubing., C., Jun., W., Shijie., H., Yumei., Z. & Xiuxiu., W. (2011) Effects of decreased rainfall on Quercus mongolica leaf ecophysiological characteristics (in Chinese with English abstract). *Chinese Journal of Ecology*, **30**, 1908-1914.

Yi-ping., Z., Chuan-sheng., W., Nai-shen., L., Li-qing., S., Xin., L. & Yu-hong., L. (2015) The response of soil temperature to experimental warming in a subtropical evergreen broad-leaved forest in Ailao Mountains Yunnan, SW China. (in Chinese with English abstract). *Chinese Journal of Ecology*, **34**, 347-351.

Yuhong., Z., Xuehong., W., Zhenxi., S., SunLei. & Xinyu., N. (2010) Effect of simulated warming on the reproductive ecology of *Carex thibetica* Franch (in Chinese with English abstract). *Ecology and Environmental Sciences*, **19**, 1783-1788.

Zhou, X., Zhang, Y., Wang, S. & Bingchang., Z. (2010) Combined effects of simulated nitrogen deposition and drought stress on growth and photosynthetic physiological responses of two annual desert plants in Junggar Basin, China (in Chinese with English abstract). *Chinese Journal of Plant Ecology*, **34**, 1394–1403.
